# Dietary *Moringa oleifera* leaf powder and xylanase supplementation as a sustainable nutritional strategy: effects on growth performance, serum biochemistry, and selected cecal bacterial populations in broiler chickens

**DOI:** 10.3389/fvets.2026.1969225

**Published:** 2026-10-14

**Authors:** Ahmed A. A. Abdel-Wareth, Mohamed A. Fawaz, Abdalla H. H. Ali, Hazem G. M. El-Sayed, Ayman A. M. Abd El-Hafez, Atef M. Saleem, Md Salahuddin, Jayant Lohakare, Hamdy A. Hassan

**Affiliations:** 1 Poultry Center, College of Agriculture, Food and Natural Resources, Prairie View A&M University, Prairie View, TX, United States; 2 Department of Animal and Poultry Production, Faculty of Agriculture, Qena University, Qena, Egypt; 3 Regional Center for Food and Feed, Agricultural Research Center, Giza, Egypt

**Keywords:** broiler chicken, cecal bacterium, growth performance, *Moringa oleifera*, serum biochemistry, sustainable nutrition, thyroid hormone, xylanase

## Abstract

**Background:**

Phytogenic feed additives and exogenous enzymes have attracted increasing interest as sustainable nutritional strategies for improving poultry production. This study evaluated the effects of dietary *Moringa oleifera* leaf powder (MLP) and xylanase, individually and in combination, on growth performance, carcass traits, serum biochemical indices, thyroid hormones, and cecal bacterial populations in broilers.

**Methods:**

A total of 160 one-day-old Cobb broiler chicks were randomly assigned to four dietary treatments in a 2 × 2 factorial arrangements consisting of two levels of MLP (0 or 4 g/kg diet) and two levels of xylanase (0 or 1.5 g/kg diet). Each treatment included five replicate pens of eight birds and was fed for 42 d. Growth performance, carcass characteristics, serum metabolites, and cecal bacterial populations were determined.

**Results:**

Dietary treatments significantly affected growth performance variables. Birds receiving the combined MLP and xylanase supplementation exhibited greater final body weight and body weight gain and lower feed conversion ratio than birds fed the control diet (*p* < 0.05). Feed intake was reduced by MLP supplementation, particularly during the grower and overall experimental periods. Relative liver weight was reduced in xylanase-supplemented birds (*p* < 0.05), whereas dressing percentage and the relative weights of other organs were not affected. Dietary treatments also influenced serum biochemical traits and thyroid hormones. MLP supplementation reduced serum glucose concentration, whereas MLP alone reduced serum total lipid concentration, whereas xylanase affected circulating thyroid hormone concentrations (*p* < 0.05). Serum total protein, albumin, globulin, and albumin-to-globulin ratio were not altered by dietary treatment. Cecal bacterial enumeration showed MLP alone increased Lactobacillus counts, whereas all supplemented treatments reduced *E. coli* counts compared with the control group (*p* < 0.05). Significant MLP × xylanase interactions were observed for biochemical variables.

**Conclusion:**

Dietary supplementation with MLP and xylanase influenced growth performance, thyroid hormone concentrations, and cultivable cecal bacterial populations in broiler chickens. The combined supplementation resulted in the most favorable growth performance and feed efficiency among the evaluated treatments. These findings support the potential application of MLP and xylanase as components of sustainable nutritional strategies in broiler production. Further studies are warranted to elucidate the biological mechanisms underlying these responses.

## 1 Introduction

Sustainable improvement in broiler production requires multifaceted strategies that enhance growth performance, feed conversion, immune status, and overall physiological health. Traditionally, antibiotic growth promoters (AGPs) were widely employed to achieve these goals. However, increasing global concern over antimicrobial resistance, combined with regulatory bans on AGPs in many countries, has created a pressing demand for natural, safe, and effective alternatives. Among the most promising candidates are phytogenic feed additives, exogenous enzymes, prebiotics, probiotics, and organic acids, all of which have shown potential in modulating gut microbiota, enhancing nutrient utilization, and supporting immune function in poultry (1–3).

*Moringa oleifera* leaves (MOL) are increasingly recognized for their phytochemical richness and nutritional density. They contain flavonoids, saponins, alkaloids, tannins, and potent antioxidants such as vitamin C, vitamin E, β-carotene, phenolic compounds, and trace elements like zinc and selenium (4). These constituents confer multiple functional benefits, including antioxidant protection, lipid-lowering effects, immune modulation, and antimicrobial activity (5). Previous studies have demonstrated that MOL supplementation in broiler diets can improve body weight gain (BWG), feed efficiency, and carcass quality while reducing serum cholesterol and triglyceride levels (6–8). MOL has also been associated with improved red blood cell counts, hemoglobin concentration, and oxidative stability (8, 9). Despite these benefits, the high content of indigestible fiber (157.5 g/kg DM) and condensed tannins in MOL can limit its utilization and reduce nutrient availability (10, 11). Specifically, non-starch polysaccharides such as cellulose, pectin, and xylan increase digesta viscosity, impair nutrient absorption, and contribute to suboptimal growth performance and poor litter quality in poultry (12, 13).

Recently, the use of exogenous enzymes in intensive animal farming has been steadily increasing due to their proven ability to reduce anti-nutritional factors in feed and enhance feed efficiency, digestibility, and nutrient bioavailability, leading to notable economic and environmental benefits (14). Numerous studies have reported that the inclusion of enzymes can significantly improve the growth performance of broiler chickens (15, 16). A wide range of exogenous enzymes such as xylanase, β-glucanase, α-galactosidase, amylase, lipase, protease, and phytase are commonly used in broiler production (17, 18). Among these, xylanase has gained particular interest as a feed additive due to its ability to degrade xylan, a major component of plant cell walls. Supplementation with xylanase in broiler diets has been shown to enhance antioxidant status (7, 19–21), stimulate immune response (22), and improve the digestibility of nutrients such as starch and crude protein, especially in wheat-based diets. These benefits are largely attributed to the enzyme’s ability to reduce intestinal viscosity, thus enhancing nutrient absorption (23, 24). Xylanase also serves as a valuable additive in energy-deficient diets, as it can increase body weight and improve feed conversion ratios (FCR) in broilers (25, 26).

Recent studies have increasingly focused on MLP as a functional feed additive in poultry production. MLP supplementation has been reported to improve broiler growth performance, feed efficiency, meat quality, and blood parameters, and to reduce serum cholesterol and triglyceride concentrations (8). MLP has also been reported to improve immune response and antioxidant status in broilers (27). In laying hens, a combination of MLP and mulberry leaf powder improved egg quality and antioxidant status while regulating lipid metabolism (28). Several functional feed ingredients, including phytogenic feed additives, probiotics, prebiotics, and organic acids, have been evaluated as sustainable alternatives to antibiotic growth promoters in poultry production. These additives have been reported to improve growth performance, feed efficiency, gut health, immune function, and antioxidant status, although the magnitude of responses varies according to additive type, inclusion level, diet composition, and management conditions (1–3, 18). Likewise, the effects of xylanase supplementation are not always consistent among studies. While improvements in nutrient digestibility, feed efficiency, gut health, and physiological responses have frequently been reported, growth performance responses may vary depending on dietary substrate composition, enzyme characteristics, and experimental conditions (21, 23–26). These variable outcomes highlight the importance of evaluating combinations of functional feed additives and exogenous enzymes in poultry diets. To our knowledge, limited information is available regarding the combined inclusion of *Moringa oleifera* leaf powder and xylanase in broiler diets. It was hypothesized that combined supplementation with MLP and xylanase would improve growth performance and feed conversion efficiency and modulate carcass traits and selected blood biochemical parameters to a greater extent than either additive provided alone. Therefore, this study evaluated the effects of individual and combined supplementation with MLP and xylanase on growth performance, carcass traits, selected serum biochemical indices, thyroid hormones, and selected cecal bacterial populations in broiler chickens.

## 2 Materials and methods

### 2.1 Collection and preparation of Moringa leaf powder

Fresh Moringa leaves were collected from trees located near the experimental area at the poultry farm of the Faculty of Agriculture, South Valley University, Qena, Egypt. The leaves were dried in an oven at 55 °C for 48 h and ground into Moringa leaf powder (MLP) using a commercial milling machine. The prepared MLP was then incorporated into the experimental diets. The bioactive compound profile of MLP was determined by GC–MS (gas chromatography–mass spectrometry) as described by Ali et al. (29).

### 2.2 Diet preparation and chemical composition

All experimental diets were formulated in accordance with the nutrient requirements for broilers recommended by the NRC (30). The ingredients were finely ground and thoroughly mixed using a horizontal feed mixer for 15 min to ensure homogeneity and uniform distribution of ingredients and additives (MLP and/or xylanase). All diets were prepared weekly in batches and stored in airtight containers at room temperature to maintain nutrient stability. The basal diet (control) was formulated for both the starter and grower phases. To test the effect of *Moringa oleifera* leaf powder (MLP), MLP was included at 4 g/kg diet. Xylanase enzyme was incorporated into the basal diet as a powder without altering its nutrient composition (Table 1). The proximate composition of the specific MLP batch used in the present experiment was not determined analytically. However, previous studies have reported that MLP contains approximately 21.9–28.1% crude protein, 4.7–5.6% ether extract, 9.3–11.0% crude fiber, and 12.0–15.2% ash on a dry matter basis (31, 32). Because MLP was included at a very low level (4 g/kg diet), replacing only small amounts of conventional ingredients, its contribution to the overall protein, lipid, energy, and other nutrient concentrations of the diets was expected to be minimal. Therefore, ingredient-specific nutrient values for MLP were not incorporated into the diet formulation calculations.

**Table 1:** Ingredient composition and analyzed chemical composition of the experimental basal diets (g/kg, as-fed basis).

Ingredient (g/kg)	Starter diet		Grower diet	
	Basal diet	MLP	Basal diet	MLP
Corn	551.50	550.50	600.00	599.00
Soybean meal (44% CP)	285.50	282.50	250.00	247.00
Corn gluten meal(60% CP)	95.50	95.50	60.00	60.00
Vitamin-mineral premix^1^	3.04	3.04	3. 00	3.00
Sunflower oil	30.00	30.00	55.20	55.20
Monocalcium phosphate	20.00	20.00	18.00	18.00
Limestone	10.00	10.00	10.00	10.00
Salt	3.04	3.04	3.80	3.80
DL-methionine	0.42	0.42	0.00	0.00
L-lysine HCl	1.00	1.00	0.00	0.00
*Moringa* Leaf Powder	0.00	4.00	0.00	4.00
Total	1,000	1,000	1,000	1,000
Chemical composition (g/kg)				
Dry matter	916.00	912.00	915.00	915.00
Crude protein	234.0	230.6	216.00	213.00
Ether extract	53.20	55.10	57.40	57.70
Crude fiber	25.10	25.80	37.90	38.20
Ash	64.30	64.50	61.70	61.80
Calcium	13.22	13.33	12.84	12.85
Available phosphorus	7.05	7.05	7.21	7.21
ME kcal/kg diet	3,000	3,005	3,220	3,223

1 The vitamin-mineral premix was included at 3.04 g/kg diet in the starter diets and 3.00 g/kg diet in the grower diets, providing the following amounts per kilogram of diet: vitamin A, 6,250,000 IU; vitamin D3, 2,500,000 IU; vitamin E, 25,000 mg; vitamin K3, 1,750 mg; vitamin B1, 500 mg; vitamin B2, 2,750 mg; vitamin B6, 1,250 mg; vitamin B12, 10 mg; niacin, 20,000 mg; calcium pantothenate, 5,000 mg; folic acid, 500 mg; biotin, 50 mg; Fe, 22,000 mg; Mn, 31,000 mg; Cu, 2,500 mg; Zn, 37,500 mg; I, 650 mg; Se, 113 mg; Co, 50 mg; ethoxyquin, 250 mg; and wheat bran carrier, 120 g. Xylanase was included at 1.5 g/kg in the designated treatments and was not considered to contribute to the calculated nutrient composition of the diets.

### 2.3 Experimental design and management

The experiment was conducted at the Experimental Poultry Farm, Department of Animal and Poultry Production, Faculty of Agriculture, South Valley University, Qena, Egypt. The care and handling of the birds and sample collection were approved by the South Valley University Animal Care and Use Committee (IACUC) under protocol SVU# 2025–0707. All experimental procedures involving birds were conducted strictly in accordance with the relevant institutional guidelines and regulations and following ARRIVE guidelines. A total of 160 unsexed day-old Cobb broiler chicks (procured from a commercial hatchery) were randomly assigned to four dietary treatments, each comprising five replicates (8 birds per replicate). Birds were housed in standard cages (100 × 50 × 45 cm) under a 24-h light regimen with an intensity of 15 Lux for the first 3 days, which was subsequently reduced to 5 Lux for the remainder of the 42-day experimental period. Feed and water were provided ad libitum throughout the experimental period. Birds were fed mash-form diets according to their assigned treatments. They were fed starter and grower diets from 1 to 21 and 22 to 42 days of age, respectively. Room temperature was initially maintained at approximately 33 °C during the first week and was gradually decreased by 2 °C per week until reaching 24 °C by the fourth week, where it was maintained until the end of the experiment period. Temperature and relative humidity were recorded daily using a thermo-hygrograph (Clock/ Humidity model HTC-2). The four dietary treatments were as follows: Control: Basal diet without any supplementation; basal diet + 4 g/kg Moringa leaf powder (MLP); basal diet + 1.5 g/kg xylanase (Xy); basal diet + a combination of basal diet + a combination of 4 g/kg MLP and 1.5 g/kg xylanase. The selection of these supplementation levels was based on previous studies that reported favorable physiological and metabolic responses of broiler chickens to MLP at 4 g/kg and xylanase at 1.5 g/kg (22, 24, 33). Only one inclusion level of each additive was evaluated, so the design did not permit assessment of dose–response relationships.

The formulation was designed to evaluate potential combined effects of the phytogenic and enzymatic components. The enzyme xylanase (AXTRA XB, Danisco Animal Nutrition, Marlborough, Wilts, UK) was included to aid in the digestion of the high-fiber diet. This endo-1,4-β-xylanase (12,200 U/g) is produced by a genetically modified strain of *Trichoderma reesei*. Based on the declared product activity and the inclusion rate of 1.5 g/kg, the calculated dietary xylanase activity was approximately 18,300 U/kg of feed. Diets were fed in mash form and were not pelleted. Dietary xylanase activity was not verified analytically, and enzyme recovery and post-manufacturing stability were not determined in the present study; these represent limitations of the enzyme characterization reported here.

### 2.4 Chemical analyses and diet composition

Diet samples were collected, oven-dried at 55 °C, and composited by weighing period for further analysis. The composite samples were ground using a 1-mm screen (Wiley mill; Philadelphia, PA, USA) and subsequently dried at 105 °C for 2 h to determine analytical dry matter (DM) content according to AOAC (34) (Method 930.15). Organic matter (OM) content was calculated as 100 minus the ash percentage, with ash determined after incineration at 500 °C for 5 h according to AOAC (34) (Method 942.05). Ether extract (EE) was determined according to AOAC (34) (Method 954.02), and crude protein (CP) was determined according to AOAC (34) (Method 984.13). Gross energy (GE) was determined using a Parr adiabatic bomb calorimeter (Moline, IL, USA). Experimental diets were formulated to meet or exceed the nutrient requirements of broiler chickens according to established recommendations (30). The starter diet (1–21 d) contained 234 g/kg crude protein (CP) and 3,000 kcal/kg metabolizable energy (ME), whereas the grower diet (22–42 d) was formulated to provide 216 g/kg CP and 3,220 kcal/kg ME shown in Table 1. Both diets had a dry matter content exceeding 910 g/kg. The diets contained moderate levels of ether extract (53.2–57.7 g/kg) and crude fiber (25.1–38.2 g/kg), ensuring adequate energy supply and gastrointestinal function. A vitamin–mineral premix was included in all diets to provide sufficient levels of essential micronutrients throughout the experimental period. The bioactive compound profile of *Moringa oleifera* leaf powder (MLP), expressed on a dry matter basis, is presented in Table 2.

**Table 2:** Bioactive compounds of *Moringa* leaf powder (MLP) on DM basis.

Bioactive compound	Content (g/kg)	Class
Quercetin	1.2	Flavonoids
Kaempferol	1.0	Flavonoids
Gallic acid	0.6	Phenolic Acids
Chlorogenic acid	0.9	Phenolic Acids
Caffeic acid	0.5	Phenolic Acids
RBITC (4-rhamnopyranosyloxybenzyl isothiocyanate)	1.1	Isothiocyanates
Moringinine	0.6	Alkaloids
Total saponins	32.3	Saponins
Total tannins	10.2	Tannins

Values are expressed on a dry matter (DM) basis and represent the mean of three analytical replicates.

### 2.5 Growth performance

Birds were individually weighed on days 1, 21, and 42 of the experimental periods. Growth performance indices were calculated throughout the experimental period. Average body weight gain was determined by subtracting the initial body weight from the final body weight and dividing the result by the number of days on feed. Total weight gain was calculated as the difference between final and initial body weights. The amount of feed offered and refused was recorded daily and feed intake (FI) was calculated by subtracting the residual feed from the amount offered. Feed conversion ratio (FCR) was expressed as the ratio of total feed intake (g) to total body weight gain (g), reflecting the efficiency with which birds converted feed into body mass,

### 2.6 Carcass traits and organ measurements

At 42 days of age, 10 birds (two per replicate) from each treatment group were randomly selected, fasted overnight with free access to water, and then slaughtered. Birds were weighed, defeathered, and eviscerated. Dressing percentage was calculated by dividing the carcass weight (after removal of head, neck, shanks, viscera, spleen, liver, gizzard, heart, and abdominal fat) by the pre-slaughter body weight. Organs including the spleen, pancreas, liver, heart, abdominal fat, and empty gizzard were weighed and expressed as percentages of live body weight. The lengths of the small intestine and cecum were also measured.

### 2.7 Serum biochemical and hormonal parameters

At the end of the experiment period, eight birds were randomly selected from each treatment group. Blood samples (2 mL) were taken from the wing vein using sterile syringes and collected into vacutainer tubes. Blood samples were allowed to clot at room temperature and then centrifuged at 3,000 rpm for 10 min to obtain serum. The separated serum was transferred into clean tubes and stored at −20 °C for further analysis. Commercial diagnostic kits (Spectrum Chemical Company, Obour City, Cairo, Egypt) were used to determine the concentrations of total protein, albumin, glucose, total cholesterol, triglycerides, and total lipids. The concentrations of thyroid hormones, triiodothyronine (T3) and thyroxine (T4), were quantified using the radioimmunoassay (RIA) technique as described by Darras et al. (35).

### 2.8 Cecal microbial enumeration

At slaughter, cecal samples were aseptically collected from each selected chicken for microbiological analysis. Each cecum was wrapped in plastic, placed in sterile bags containing 50 mL of ice-cold cryoprotective broth (36), and stored at −80 °C until analysis (37). Prior to examination, the frozen ceca were thawed for 20 min and removed from their storage bags. The contents of each cecum were then aseptically transferred into new sterile bags. These contents were diluted tenfold (10% wt/vol) in sterile, ice-cold anoxic phosphate-buffered saline (PBS, 0.1 M, pH 7.0) and homogenized for 3 mins. The resulting digesta slurries were serially diluted using 1 mL of PBS, from 10^−1^ to 10^−7^.

For microbial enumeration, duplicate samples were plated on selective agar media: M. R. S. for *Lactobacillus* spp., MacConkey agar for *Escherichia coli*, along with media suitable for total bacterial counts (38). Plates were incubated at 37 °C for 48 h, after which colony-forming units (CFUs) were enumerated, with up to 120 colonies counted per plate, as described by Fawaz et al. (39).

### 2.9 Statistical analysis

All data were analyzed using a completely randomized design (CRD) with the General Linear Model (GLM) procedure of SAS software (version 9.2; SAS Institute Inc., Cary, NC, USA) (40). Dietary treatment was included as a fixed effect, whereas pen served as the experimental unit and was considered a random effect. Growth performance and feed intake variables were measured and analyzed at the pen (replicate) level, with five pens per treatment. Carcass traits, serum variables, and cecal bacterial counts were obtained from individual birds sampled within pens, as described above. Data were analyzed as a 2 × 2 factorial arrangement of MLP (0 or 4 g/kg) and xylanase (0 or 1.5 g/kg), with both main effects and their interaction included in the model. When significant differences were detected, means were separated using Duncan’s multiple range test. Statistical significance was declared at *p* < 0.05, whereas differences at 0.05 < *p* ≤ 0.10 were considered trends. Normality of residuals was assessed by the Shapiro–Wilk test and homogeneity of variances by Levene’s test.

## 3 Results

### 3.1 Growth performance

Dietary treatments significantly affected growth performance throughout the experimental period (Table 3). At 21 d, broilers fed the combined MLP and xylanase diet exhibited the highest body weight (862.5 g), followed by the control (829.2 g), MLP (820.8 g), and xylanase (816.7 g) groups (*p* < 0.001). A similar pattern was observed at 42 d, with birds receiving the combined treatment achieving the greatest final body weight (2135.4 g), whereas MLP- and xylanase-fed birds showed lower body weights than the control group (*p* < 0.001). Body weight gain (BWG) was significantly affected by dietary treatment during the starter, grower, and overall periods (*p* < 0.001). Birds fed the combined MLP and xylanase diet exhibited the highest BWG during 1 to 21 d, 21 to 42 d, and 1 to 42 d. In contrast, broilers receiving MLP or xylanase alone showed lower BWG than the control group during all growth phases. Feed intake (FI) differed among treatments. During the starter phase, FI was lower in the MLP group than in the other treatments, whereas no differences were observed among the control, xylanase, and combined-treatment groups. During the grower and overall periods, birds fed MLP alone or in combination with xylanase consumed less feed than control birds, with intermediate values observed for the xylanase group (*p* < 0.001). Feed conversion ratio (FCR) was also affected by dietary treatment. During the starter phase, the combined treatment resulted in a lower FCR than all other groups (*p* < 0.001). During the grower period, birds receiving the combined treatment had the lowest FCR, while the control group exhibited the highest value. Over the entire experimental period, FCR was reduced in all supplemented groups relative to the control, with the lowest value observed in birds receiving both MLP and xylanase (*p* < 0.001).

**Table 3:** Effects of *Moringa* leaf powder (MLP), xylanase and their combination on productive performance of broiler chicken.

Item	Treatment				SEM	*p*-value		
	Control	MLP	Xy	MLP + Xy		MLP	Xy	MLP × Xy
Body weight, (g/bird)								
1 day	44.000	45.400	44.600	44.300	0.400	0.185	0.528	0.120
21 days	829.2^b^	820.8^c^	816.7^d^	862.5^a^	1.300	<0.001	<0.001	<0.001
42 days	2064.6^b^	2026.2^c^	2028.0^c^	2135.4^a^	4.600	<0.001	<0.001	<0.001
Body weight gain, (g/bird)								
1–21 days	785.2^b^	775.4^bc^	772.1^c^	818.2^a^	3.510	<0.001	0.003	<0.001
21–42 days	1235.4^b^	1205.4^c^	1211.3^c^	1272.9^a^	4.810	0.011	0.002	<0.001
1–42 days	2020.6^b^	1980.8^c^	1983.4^c^	2091.2^a^	2.920	<0.001	<0.001	<0.001
Feed intake, (g/bird)								
1–21 days	1210.8^a^	1195.5^b^	1207.5^a^	1206.5^a^	1.910	0.003	0.078	0.002
21–42 days	2783.3^a^	2522.4^c^	2627.1^b^	2520.2^c^	7.410	<0.001	<0.001	<0.001
1–42 days	3994.1^a^	3717.9^c^	3834.6^b^	3726.4^c^	11.500	<0.001	<0.001	<0.001
Feed conversion ratio								
1–21 days	1.543^a^	1.542^a^	1.565^a^	1.476^b^	0.010	0.002	0.048	0.002
21–42 days	2.254^a^	2.093^bc^	2.173^ab^	1.984^c^	0.035	0.001	0.027	0.697
1–42 days	1.977^a^	1.877^c^	1.934^b^	1.782^d^	0.009	<0.001	<0.001	0.020

^a–c^Means within the same row bearing different superscript letters differ significantly (*p* < 0.05). SEM = standard error of the mean. Control = basal diet; MLP = basal diet + *Moringa oleifera* leaf powder at 4 g/kg; Xy = basal diet + xylanase at 1.5 g/kg; MLP + Xy = basal diet + *M. oleifera* leaf powder at 4 g/kg and xylanase at 1.5 g/kg.

### 3.2 Carcass characteristics

Carcass characteristics are presented in Table 4. Dressing percentage and the relative weights of the gizzard, heart, spleen, and pancreas were not significantly influenced by treatment (*p* > 0.05). Likewise, intestinal and cecal lengths did not differ among treatments (*p* > 0.05). Relative liver weight was significantly affected by dietary treatment (*p* = 0.013). Birds fed xylanase alone or in combination with MLP exhibited lower liver weights than those in the control group, whereas broilers fed MLP alone showed intermediate values and did not differ significantly from the control.

**Table 4:** Effects of *Moringa* leaf powder (MLP), xylanase and their combination on productive performance of broiler chicken.

Item	Treatment				SEM	*p*-values		
	Control	MLP	Xy	MLP + Xy		MLP	Xy	MLP × Xy
Carcass characteristics percentage								
Dressing, %	79.135	78.956	81.263	78.446	0.693	0.705	0.918	0.552
Liver, %	2.756^a^	2.424^ab^	1.961^bc^	1.876c	0.127	0.741	0.023	0.013
Gizzard, %	1.120	1.350	1.195	1.209	0.065	0.124	0.230	0.714
Heart, %	0.522	0.466	0.439	0.414	0.020	0.065	0.084	0.263
Spleen, %	0.141	0.152	0.110	0.128	0.009	0.210	0.340	0.482
Pancreas, %	0.220	0.197	0.169	0.185	0.009	0.075	0.095	0.286
Intestine length, cm	181.0	191.7	124.0	206.3	16.3	0.084	0.075	0.332
Cecum length, cm	17.70	18.70	17.30	17.00	0.60	0.069	0.214	0.827

^a–c^Means within the same row bearing different superscript letters differ significantly (*p* < 0.05). SEM = standard error of the mean. Control = basal diet; MLP = basal diet +*Moringa oleifera* leaf powder at 4 g/kg; Xy = basal diet + xylanase at 1.5 g/kg; MLP + Xy = basal diet + *M. oleifera* leaf powder at 4 g/kg and xylanase at 1.5 g/kg.

### 3.3 Blood parameters

#### 3.3.1 Serum thyroid hormone concentrations

Dietary supplementation significantly influenced serum thyroid hormone concentrations in broiler chickens (*p* ≤ 0.005; Figure 1). As illustrated in Figure 1, serum thyroxine (T4) concentration was significantly reduced (*p* < 0.05) in broilers fed the xylanase-supplemented diet compared to those in the control and MLP groups. Birds receiving the combined MLP and xylanase treatment exhibited T4 levels comparable to the control and MLP-fed broilers. Similarly, dietary treatment showed a significant effect on serum triiodothyronine (T3) concentrations (*p* < 0.001; Figure 1). Broilers fed the control and MLP diets showed the highest serum T3 levels and did not differ (*p* > 0.05) from each other. In contrast, supplementation with xylanase alone resulted in significantly lower (*p* < 0.05) T3 concentration among all groups. The combined MLP and xylanase group also showed reduced T3 concentrations, which did not differ significantly from the xylanase-only group but were lower (*p* < 0.05) than the control and MLP groups.

**Figure 1:**
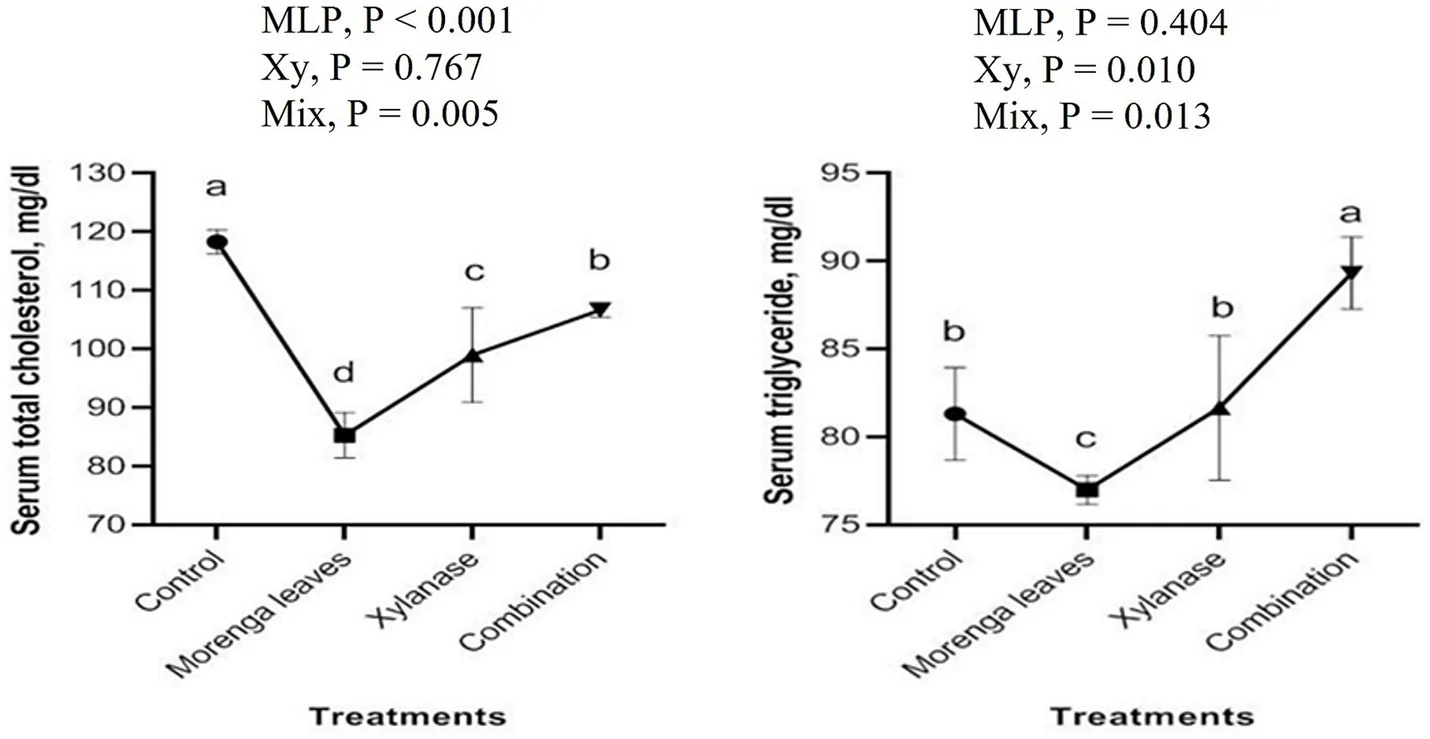
Effects of dietary treatments on serum thyroid hormone concentrations in poultry. Serum thyroxine (T4) concentration and serum triiodothyronine (T3) concentration in birds fed the control diet, a diet supplemented with *Moringa oleifera* leaf powder (MLP), xylanase, or a combination of MLP and xylanase. Dietary treatment affected serum T4 (*p* = 0.005) and T3 (*p* < 0.001) concentrations. Values are presented as means ± SEM. Means within each panel with different superscript letters (a–c) differ significantly (*p* < 0.05).

#### 3.3.2 Serum lipid profile

Dietary treatments significantly affected serum lipid metabolites in broiler chickens (*p* < 0.001; Figure 2). Birds fed the control diet exhibited the lowest serum cholesterol concentrations, whereas supplementation with MLP resulted in the highest values (*p* < 0.05). Birds receiving xylanase alone or the combination showed intermediate cholesterol concentrations, significantly higher than the control but lower than the MLP group (*p* < 0.05). The MLP × xylanase interaction for serum cholesterol was significant (*p* < 0.01). Similarly, serum triglyceride concentrations were significantly influenced by dietary treatment (*p* = 0.011; Figure 2). Broilers fed the MLP diet exhibited the numerically lowest serum triglyceride concentrations, which did not differ significantly from the control or xylanase groups (*p* > 0.05). In contrast, the combined MLP and xylanase treatment resulted in the highest serum triglyceride concentration, which was significantly greater (*p* < 0.05) than all other dietary groups.

**Figure 2:**
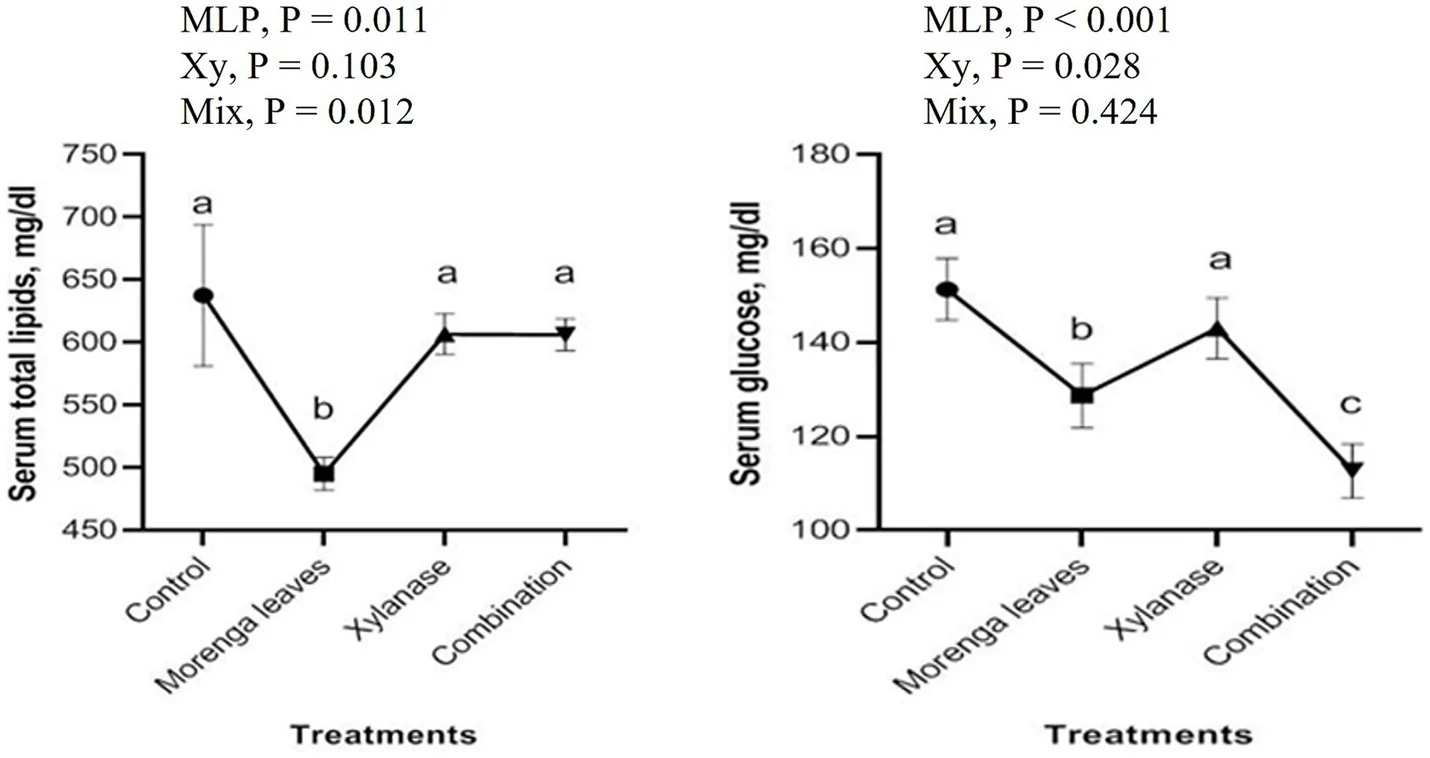
Effects of dietary treatments on serum lipid profile in broiler chickens. Serum total cholesterol concentration and serum triglyceride concentration in broilers fed a basal control diet, *Moringa oleifera* leaf powder (MLP), xylanase, or a combination of MLP and xylanase. Dietary treatment influenced serum cholesterol (*p* < 0.001) and triglyceride (*p* = 0.011) concentrations. Values are expressed as means ± SEM. Bars with different superscript letters (a–d) within each panel are significantly different (*p* < 0.05).

#### 3.3.3 Serum total lipids and glucose concentrations

Dietary supplementation had a significant effect on serum total lipids and glucose concentrations in broiler chickens (*p* ≤ 0.008; Figure 3). As shown, serum total lipid concentration was significantly reduced (*p* < 0.05) in broilers fed MLP compared with all other groups. Broilers fed the control, xylanase, and combined MLP and xylanase diets exhibited higher and comparable serum total lipid concentrations (*p* > 0.05). Serum glucose concentrations were also affected by dietary treatment (*p* = 0.002). Broilers fed the control and xylanase-supplemented diets exhibited similar serum glucose concentrations (*p* > 0.05). In contrast, dietary inclusion of MLP significantly reduced serum glucose levels (*p* < 0.05) compared to the control group. The lowest serum glucose concentration was observed in broilers receiving the combined MLP and xylanase diet, which was significantly lower (*p* < 0.05) than all other groups.

**Figure 3:**
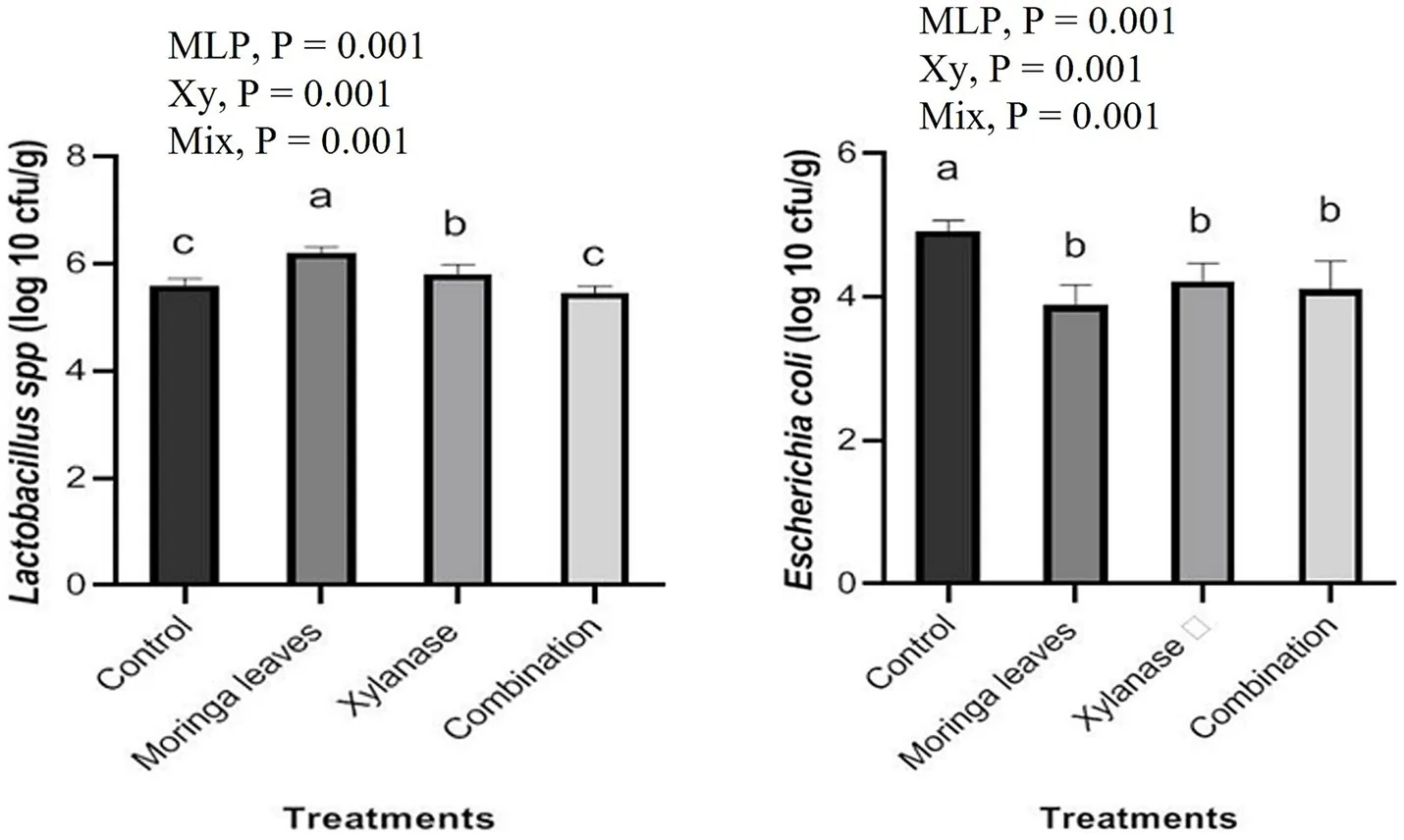
Effects of dietary treatments on serum total lipids and glucose concentrations in broiler chickens. Serum total lipid concentration and serum glucose concentration in broilers fed a basal control diet, a diet supplemented with *Moringa oleifera* leaf powder (MLP), xylanase, or a combination of MLP and xylanase. Dietary treatment affected serum total lipids (*p* = 0.008) and glucose (*p* = 0.002) concentrations. Values represent means ± SEM. Means with different superscript letters (a,b) within each panel differ significantly (*p* < 0.05).

#### 3.3.4 Serum protein profile

Serum protein concentrations and the albumin-to-globulin (A/G) ratio are presented in Figure 4. Serum total protein concentration was not significantly affected by dietary treatment (*p* = 0.532). Birds fed diets supplemented with MLP or xylanase showed numerically higher serum total protein concentrations compared to the control group, whereas broilers receiving the combined MLP and xylanase diet showed the numerically lowest values. Similarly, serum albumin concentration was not significantly affected by dietary treatments (*p* = 0.366). Although broilers fed the xylanase-supplemented diet displayed numerically higher albumin levels, no significant differences (*p* > 0.05) were detected among treatments.

**Figure 4:**
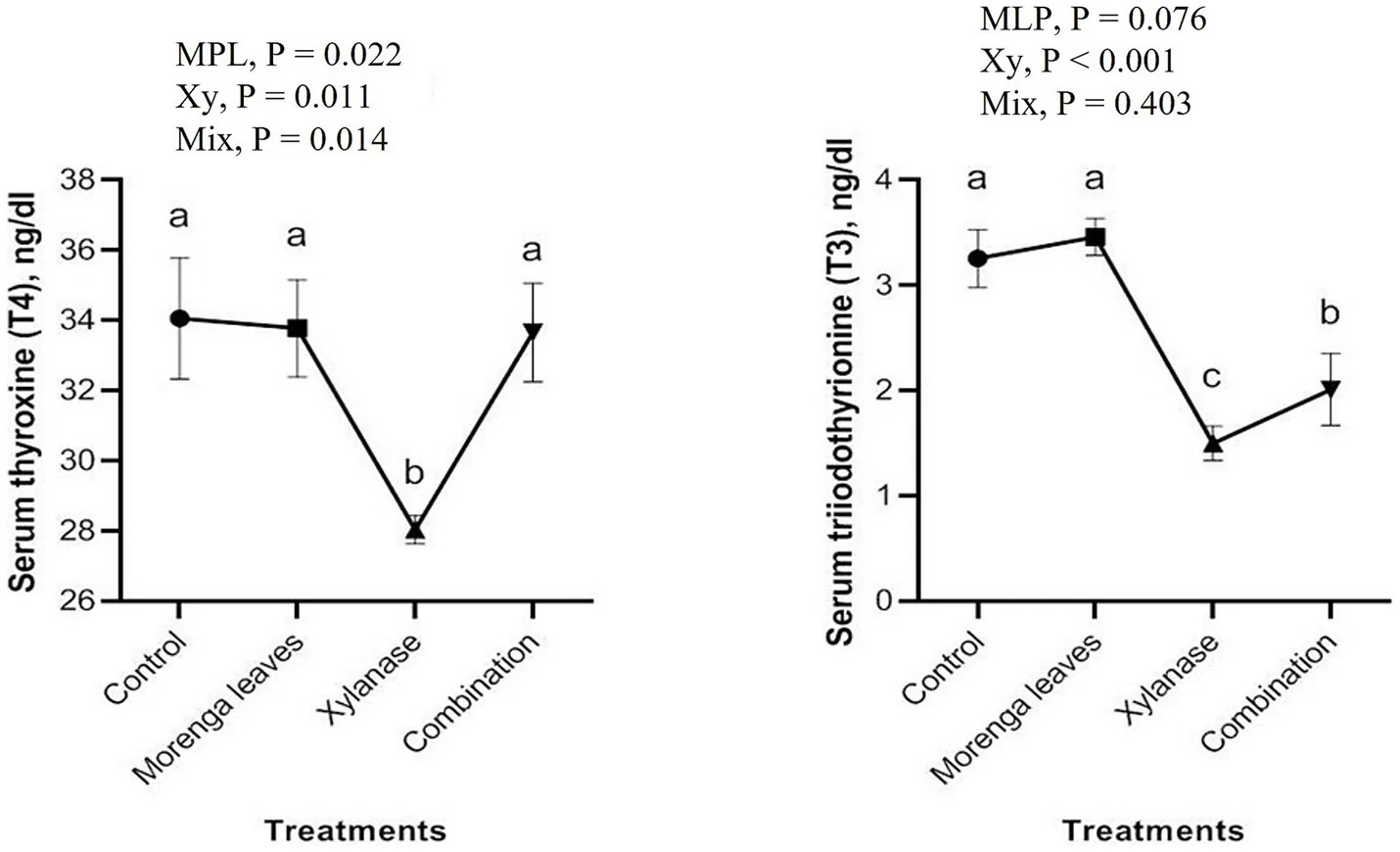
Effects of dietary treatments on serum protein fractions and albumin-to-globulin ratio in broiler chickens. Serum total protein concentration, serum albumin concentration, serum albumin-to-globulin (A/G) ratio, and serum globulin concentration in broilers fed a basal control diet, *Moringa oleifera* leaf powder (MLP), xylanase, or a combination of MLP and xylanase. Dietary treatment did not significantly affect serum total protein (*p* = 0.532), albumin (*p* = 0.366), A/G ratio (*p* = 0.589), or globulin concentration (*p* = 0.688). Values are presented as means ± SEM. No superscript letters are shown, as no treatment differences were significant.

The serum albumin-to-globulin ratio did not differ significantly among dietary groups (*p* = 0.589). The A/G ratio was numerically highest in broilers fed xylanase (1.553) and the control diet (1.547), intermediate with MLP (1.509), and lowest with the combined treatment (1.315). Serum globulin concentration was also not significantly affected by dietary treatment (*p* = 0.688). Broilers fed the combined MLP and xylanase diet exhibited the numerically highest serum globulin concentration. No treatment differed significantly from the control for any serum protein fraction.

### 3.4 Selected cecal bacterial populations

Dietary supplementation significantly influenced the cultivable cecal bacterial populations enumerated in broiler chickens (*p* < 0.001; Figure 5). The population of *Lactobacillus* spp. differed significantly among groups. Broilers fed the diet supplemented with MLP exhibited the highest *Lactobacillus* counts (*p* < 0.05). Birds receiving the xylanase-supplemented diet showed intermediate *Lactobacillus* populations, which were significantly higher than those observed in the control and combined MLP and xylanase groups but lower than the MLP group (*p* < 0.05). No significant difference (*p* > 0.05) was observed between the control and combined treatment groups for *Lactobacillus* counts. Conversely, counts of *Escherichia coli* were significantly affected by dietary treatment (*p* < 0.001). Broilers fed the control diet exhibited the highest cecal *E. coli* counts. Supplementation with MLP resulted in a significant reduction (*p* < 0.05) in *E. coli* counts compared to the control. Similarly, birds receiving xylanase alone or in combination with MLP also showed significantly lower *E. coli* populations than the control group (*p* < 0.05), with no significant differences among these three supplemented treatments.

**Figure 5:**
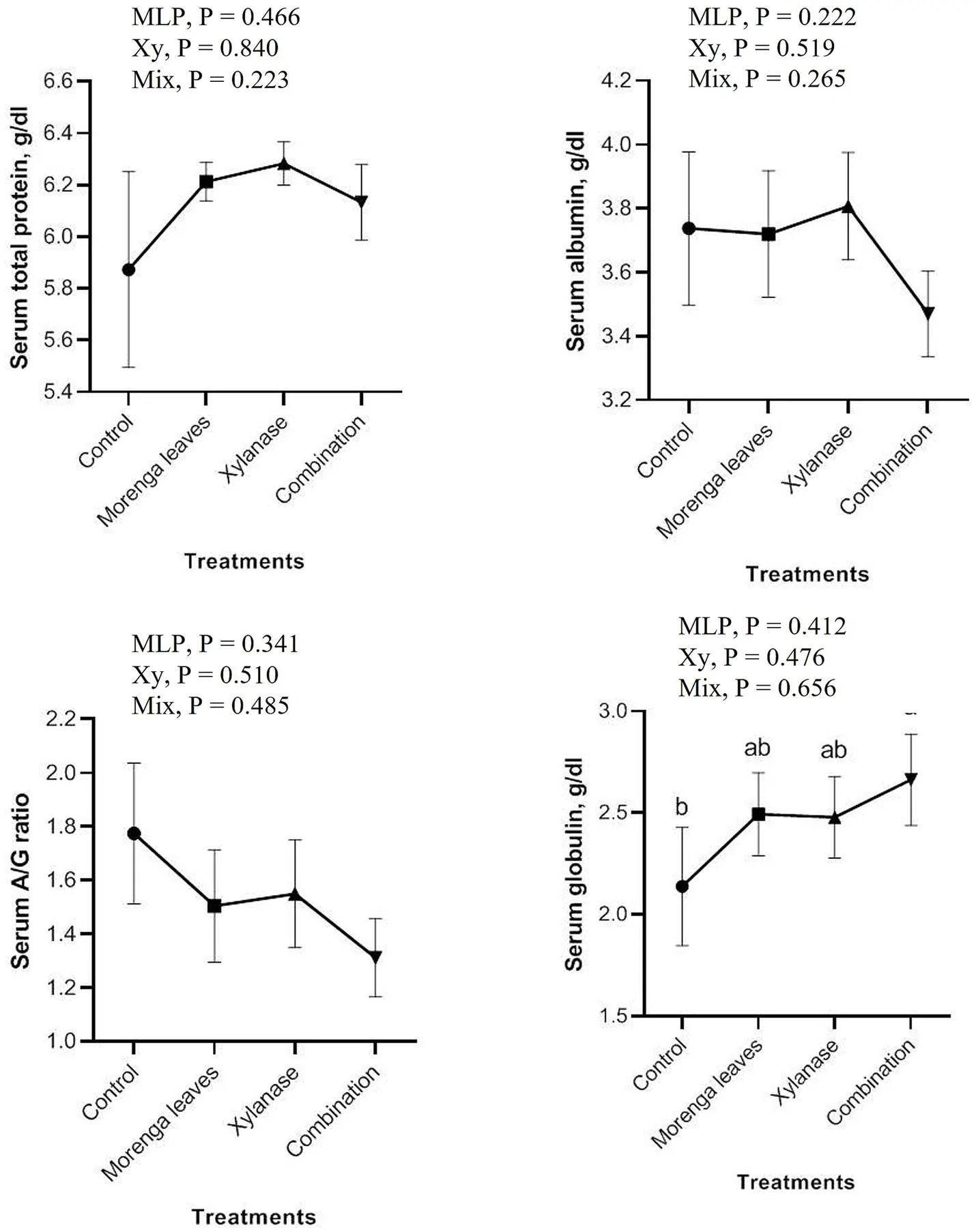
Effects of dietary treatments on intestinal microbial populations in broiler chickens. *Lactobacillus* spp. counts and *Escherichia coli* counts (log₁₀ cfu/g) in broilers fed a basal control diet, *Moringa oleifera* leaf powder (MLP), xylanase, or a combination of MLP and xylanase. Dietary treatment significantly affected intestinal microbial populations (*p* < 0.001). Values are expressed as means ± SEM. Bars with different superscript letters (a–c) within each panel differ significantly (*p* < 0.05).

## 4 Discussion

The present study evaluated the individual and combined effects of MLP and xylanase supplementation on growth performance, carcass traits, blood biochemistry, and selected cecal bacterial populations in broiler chickens. Both additives, and particularly their combined inclusion, were associated with improved growth performance and with changes in selected metabolic indices and cecal bacterial counts. *Moringa oleifera* leaves have been proposed as a promising feed additive for poultry due to their strong antimicrobial, nutritional, and immunomodulatory properties (41–44). The improvement in BW and BWG was most pronounced with the MLP and xylanase combination. This is consistent with a greater response to the combined inclusion of a plant-based additive and an exogenous enzyme; the MLP × xylanase interaction was tested statistically in the 2 × 2 factorial arrangement described in the Materials and methods. The MLP, rich in essential amino acids, antioxidants (e.g., vitamin C, flavonoids), and bioavailable minerals, may have contributed to improved nutrient utilization, although neither nutrient digestibility nor immune function was measured in the present study (8, 45). Similar improvements in BWG and feed efficiency have been reported with MLP inclusion at 1–5% in broiler diets (7, 46). The greatest improvements in growth performance and feed conversion ratio were observed in the combination group; the MLP × xylanase interaction was significant for body weight, body weight gain, feed intake, and feed conversion ratio. The responses may be partially explained by mechanisms reported previously, whereby xylanase degrades fibrous cell walls, reduces digesta viscosity, and releases encapsulated nutrients. Xylanase may improve nutrient utilization through the hydrolysis of specific xylan-containing polysaccharides and reduction of digesta viscosity; however, the bioavailability of MLP bioactive compounds was not evaluated in the present study. Flavonoids and phenolics have been reported to inhibit *E. coli* and to support *Lactobacillus* spp., which is consistent with the shifts in cecal bacterial counts observed here. Any effects on oxidative status, nutrient absorption, and intestinal integrity remain speculative in the present study, because these variables were not measured. The reductions in serum lipids and glucose could be related to MLP constituents previously reported to inhibit enzymes involved in cholesterol synthesis and carbohydrate digestion, although no enzyme activities were assessed in the present study. Overall, birds receiving both supplements showed the greatest improvements in growth and feed efficiency, but the biological basis of this response cannot be established from the present data. The current findings corroborate previous studies that have explored the effect of *Moringa oleifera* leaf powder (MLP) on broiler performance. For instance, broilers fed diets containing 1.2% MLP demonstrated a significant improvement in final body weight compared to the control group (*p* < 0.05) (46). Similarly, Alabi et al. (47) reported that administering 90 mL/L of *M. oleifera* leaf extract in drinking water reduced feed intake by 12.83%, while simultaneously improving feed efficiency by 9.11%. Consistent with this, Abu Hafsa et al. (48) observed that a 1% dietary inclusion of MLP yielded the highest final body weight and optimal feed conversion ratio (FCR); however, higher inclusion levels (5%) had adverse effects on daily weight gain and FCR. In Japanese quail, dietary levels of MLP above 25 g/kg were associated with impaired growth performance and reduced feed utilization efficiency (49), possibly due to the high fiber or anti-nutritional factor content. Feed intake was reduced with MLP inclusion during all experimental phases. Similarly, Onunkwo and George (50) documented decreased feed consumption following MLP supplementation. However, El-Tazi (51) reported a significant increase in feed intake with MLP inclusion up to 5% (*p* < 0.05). These discrepancies may be attributed to differences in MLP preparation methods, phytochemical composition, or bird genetics and management conditions.

In the present study, supplementation with 1.5 g/kg of xylanase alone reduced final body weight and body weight gain, lowered feed intake, and improved feed conversion ratio relative to the control (*p* < 0.001), although the responses were smaller than those of the combination; previous work has likewise reported limited efficacy of xylanase at similar inclusion levels (24). Although xylanase has been reported to enhance nutrient digestibility by hydrolysing non-starch polysaccharides and reducing intestinal viscosity (52), it is important to note that xylanase efficacy may be substrate-dependent, and its effectiveness may vary depending on dietary composition and experimental conditions. Notably, performance benefits have been more consistently observed when xylanase is combined with other enzymes or incorporated into specific dietary substrates such as corn-based diets (53, 54). Interestingly, the current study found that combining xylanase with Moringa leaf powder (MLP) resulted in significantly improved FCR across all experimental growth phases, suggesting that its benefits were more pronounced in combination with MLP. Therefore, while xylanase alone offered limited benefits under the present conditions, its co-application with bioactive additives such as MLP may support improved growth performance in broilers.

In this study, neither MLP nor xylanase supplementation had a detrimental effect on carcass yield, as evidenced by the absence of significant differences in dressing percentage and most organ weights. This may indicate that neither additive impaired organ development at the inclusion levels tested, although organ histology and hepatic function were not assessed in the present study. However, the significant reduction in relative liver weight observed in the enzyme and combination groups could be related to an altered hepatic metabolic load, although indicators of hepatic function, inflammation, and nutrient metabolism were not measured in the present study. The MLP × xylanase interaction was not significant for relative liver weight (*p* = 0.458), indicating a xylanase main effect (*p* = 0.003) rather than an interactive response. Polyphenols and flavonoids in MLP have been reported previously to exert hepatoprotective and metabolic regulatory effects, which could be related to the present liver weight response; however, no indicator of hepatic function or metabolism was measured in this study. Although the precise mechanisms underlying this response remain to be elucidated, the findings are in line with previous reports indicating reduced liver weights following enzyme supplementation without negative impacts on function (55).

In contrast, several studies have reported no significant changes in organ weights, including liver, heart, gizzard, and abdominal fat, after xylanase supplementation at varying levels (0.005–0.01% or 200 mg/kg) (21, 54, 56). Similarly, Onunkwo and George (50) observed no meaningful differences in carcass characteristics across treatment groups. Furthermore, the unchanged lengths of the small intestine and cecum observed in the present study indicate that the performance responses were not accompanied by changes in gut length; because nutrient digestibility and intestinal morphology were not evaluated, the basis of these responses cannot be determined from the present data. Overall, the combined inclusion of MLP and xylanase did not compromise carcass quality or internal organ development.

Blood biochemical parameters are widely used as sensitive indicators of an animal’s physiological and metabolic state, often reflecting factors such as age, nutritional status, environmental conditions, and endocrine activity. Among these, the thyroid hormones triiodothyronine (T3) and thyroxine (T4) serve as reliable biomarkers for assessing thyroid gland function. T3 is considered the more biologically active hormone, with greater metabolic potency and a faster mode of action compared to T4 (57). In the present study, broilers supplemented with xylanase alone exhibited the lowest serum T3 and T4 concentrations, whereas the combination group showed similarly reduced T3 concentrations and T4 concentrations comparable to those of the control and MLP groups. This reduction suggests that xylanase may influence thyroid hormone metabolism, potentially by modulating nutrient availability or altering intestinal microbiota, which in turn could impact endocrine function. Lower circulating concentrations could also be related to altered peripheral hormone sensitivity. These interpretations remain speculative in the present study, because thyroid function, peripheral hormone sensitivity, and metabolic rate were not assessed. Interestingly, these findings partially contrast with previous reports indicating that MLP supplementation may stimulate thyroid activity, highlighting the potential for context-dependent endocrine effects. This discrepancy between studies underscores the complexity of the interactions between dietary components and endocrine function, which may vary by species, physiological status, or environmental conditions. Limited studies have directly investigated the hormonal effects of xylanase or its interaction with phytogenic additives like MLP. Some evidence suggests that multi-enzyme supplementation can increase T3 levels, as observed by Khodaei et al. (58) in laying hens. These varying responses suggest that enzyme effects on the endocrine system are highly dose and context dependent. Nonetheless, further research is warranted to clarify the mechanisms underlying these hormonal shifts and their implications for broiler growth and metabolism.

Elevated serum levels of lipids, including total cholesterol, HDL, LDL, and triglycerides, are well-established risk factors for cardiovascular dysfunction and metabolic disorders in both animals and humans. In the present study, broilers fed diets supplemented with MLP, xylanase, or their combination exhibited significantly higher serum total cholesterol concentrations than the control group. Broilers receiving MLP alone showed the lowest serum total lipid concentrations (*p* < 0.05), whereas serum triglycerides did not differ significantly from the control. The reduction in total lipids may be associated with bioactive phytochemicals in MLP, such as saponins, flavonoids, and polyphenols, which have been reported to influence lipid metabolism by inhibiting cholesterol synthesis, enhancing bile acid excretion, or reducing intestinal lipid absorption (59), none of which were measured in the present study. The concurrent increase in serum cholesterol in the MLP-fed groups is not consistent with this mechanism and warrants further investigation. Interestingly, the combination of xylanase and MLP resulted in elevated serum triglycerides compared to MLP alone, which could be related to increased dietary energy availability through fiber degradation; this explanation remains speculative, as energy utilization was not measured.

In contrast to the present cholesterol response, Essien et al. (55) observed significant reductions in total cholesterol, HDL, LDL, and triglycerides in broilers fed 2.5%, 5%, and 7.5% MLP. Similar hypolipidemic trends have also been documented in broilers supplemented with 750–1,500 ppm MLP (60, 61), supporting the lipid-modulating properties of MLP. In contrast, xylanase alone did not significantly alter serum lipid parameters in the current study, aligning with previous reports suggesting that enzyme supplementation may have limited impact on lipid profiles unless combined with other bioactive components. Nevertheless, several studies have documented the hypocholesterolemic effect of xylanase in specific contexts. For example, El-Sayed (56) reported reduced plasma cholesterol and triglyceride levels in broilers fed 200 mg/kg xylanase, while xylanase was also shown to lower total cholesterol in broilers on low-energy diets (9). These effects have been attributed previously to changes in digestion, digesta viscosity, and gut microbial composition; because digestibility, digesta viscosity, and microbial composition were not measured in the present study, this explanation could not be evaluated here.

These results indicate that MLP, and to a lesser extent xylanase, modified serum lipid concentrations in broilers. The combination of the two additives did not further reduce serum lipid concentrations relative to MLP provided alone.

Glucose serves as the primary carbohydrate and a crucial energy source for most body cells, playing a central role in metabolic processes. Previous studies have demonstrated the anti-diabetic potential of *Moringa oleifera*, where its supplementation led to significant reductions in blood glucose levels in rodent models (62, 63). In the present study, the glucose-lowering effect of MLP, observed both alone and in combination with xylanase, aligns with its reported anti-diabetic properties. Bioactive compounds in *Moringa oleifera* leaf may modulate glucose metabolism by inhibiting α-amylase and α-glucosidase enzymes, thereby reducing postprandial glucose levels (64–66); these enzyme activities were not measured in the present study. The lower glucose concentration in the combination group could be related to altered nutrient partitioning, although gut function was not assessed in the present study. Reductions in digesta viscosity and increased fiber degradation following xylanase supplementation have been reported previously and could be related to this response; digesta viscosity, fiber degradation, and nutrient absorption were not measured in the present study. Overall, dietary inclusion of MLP, alone or combined with xylanase, was associated with lower serum glucose concentrations in broilers.

Serum proteins, particularly globulins, play a crucial role in immune function and protein metabolism in poultry. In the current study, serum globulin concentration was numerically highest in broilers fed the combination of MLP and xylanase, but the effect of dietary treatment was not statistically significant. Neither the main effects nor the MLP × xylanase interaction was significant for serum globulin. Globulins include immunoglobulins (antibodies), acute-phase proteins, and other transport molecules that are vital for defending the host against pathogens and maintaining immune surveillance. Because this difference was not statistically significant, it should not be interpreted as a treatment effect. Bioactive compounds in MLP, such as flavonoids, polyphenols, and vitamin C, have been reported to possess antioxidant, antimicrobial, and immunomodulatory properties (67, 68). These phytochemicals may stimulate the immune system by enhancing lymphocyte proliferation, macrophage activity, or immunoglobulin synthesis (69). Xylanase has also been reported to modify gut microbial populations, which contribute to mucosal and systemic immunity. However, digesta viscosity, nutrient absorption, and immune function were not measured in the present study. The present data do not demonstrate such an effect, because no serum protein fraction differed significantly among treatments (70, 71). The absence of significant changes in total serum protein, albumin, and albumin-to-globulin (A/G) ratio across treatments suggests that overall protein homeostasis was not disrupted by the dietary treatments (72).

These results indicate that serum protein fractions were not significantly altered by MLP or xylanase supplementation. Because immune function was not measured directly, no conclusion regarding immune status can be drawn from the present data.

Gut microbiota plays a pivotal role in the health and productivity of broiler chickens by influencing nutrient digestion, immune development, and intestinal integrity. In the present study, MLP alone produced the highest *Lactobacillus* counts, whereas *Escherichia coli* counts were reduced by MLP, xylanase, and their combination; *Lactobacillus* counts in the combination group did not differ from the control. These changes in selected cecal bacterial populations did not follow a consistent pattern for combined inclusion, and interaction effects were not tested. These changes may be related to the antimicrobial properties of Moringa extracts and to the prebiotic effects of fiber-derived oligosaccharides reported previously; neither antimicrobial activity nor fiber degradation was measured in the present study. The microbial assessment was limited to culture-based enumeration of selected bacterial groups and therefore does not represent the entire cecal microbial community.

The antimicrobial properties of *Moringa oleifera* have been well documented and are largely attributed to its rich content of bioactive compounds such as isothiocyanates, flavonoids, tannins, and alkaloids (73, 74). These compounds have been reported to inhibit the growth of gram-negative bacteria such as *E. coli* by disrupting cell wall synthesis, altering membrane permeability, or interfering with microbial enzyme systems. Concurrently, MLP has been shown to support the proliferation of beneficial microbes such as *Lactobacillus* spp., which play a vital role in maintaining gut barrier function, competitive exclusion of pathogens, and production of short-chain fatty acids (SCFAs). Xylanase has been reported to break down complex non-starch polysaccharides into smaller fermentable oligosaccharides. These released substrates can act as prebiotics, selectively stimulating the growth of beneficial bacteria. Improved fiber degradation has also been reported to reduce digesta viscosity and to support a more stable microbial environment (75); digesta viscosity, intestinal inflammation, and nutrient utilization were not measured in the present study.

The observed reduction in *E. coli* and increase in *Lactobacillus* spp. indicate that the dietary treatments altered the cultivable cecal bacteria enumerated. Whether these changes contributed to the observed growth responses cannot be established from the present data. The ability of natural feed additives to influence gut microbial populations supports further evaluation of their potential as alternatives to antibiotic growth promoters, although no antibiotic growth promoter was included as a comparator in the present study.

Further studies employing advanced microbiome sequencing techniques are recommended to provide deeper insights into the specific microbial shifts induced by MLP and xylanase supplementation, as well as their functional consequences on host metabolism and disease resistance.

## 5 Conclusion

Dietary supplementation with *Moringa oleifera* leaf powder and xylanase influenced growth performance, selected serum biochemical parameters, thyroid hormone concentrations, and cultivable cecal bacterial populations in broiler chickens. The combined supplementation was associated with improved final body weight and feed conversion ratio, while MLP was associated with changes in serum lipid and glucose concentrations and selected cecal bacterial counts. However, the underlying mechanisms require further investigation because nutrient digestibility, intestinal morphology, digestive enzyme activity, antioxidant status, and immune responses were not evaluated in the present study.

## Data Availability

The original contributions presented in the study are included in the article/supplementary material, further inquiries can be directed to the corresponding authors.
